# A Phenotype‐Driven Discovery of Pro‐Revascularization Chalcone Derivatives Using Zebrafish and CAM Models

**DOI:** 10.1155/jt/6939974

**Published:** 2026-07-12

**Authors:** Yau-Hung Chen, Biswajit Mohanty, Tao-Sheng Li, Sheng-Fen Tung, Chi-Chung Wen, Parthapratim Munshi, Chih-Hsin Chen, Ching-Yuh Chern

**Affiliations:** ^1^ Department of Chemistry, Tamkang University, New Taipei City, Taiwan, tku.edu.tw; ^2^ Department of Chemistry, Shiv Nadar Institution of Eminence, Delhi-NCR, Greater Noida, India; ^3^ Department of Stem Cell Biology, Atomic Bomb Disease Institute, Nagasaki University, Nagasaki, Japan, nagasaki-u.ac.jp; ^4^ Institute of Public Health, National Yang Ming Chiao Tung University, Taipei, Taiwan, cam.ac.uk; ^5^ Department of Applied Chemistry, National Chia-Yi University, Chiayi, Taiwan, ncyu.edu.tw

**Keywords:** chalcone, chick, pro-angiogenic, real-time PCR, SAR, zebrafish

## Abstract

Angiogenesis is crucial for tissue repair and the treatment of ischemic diseases, yet effective small‐molecule proangiogenic agents are lacking in clinical practice. Chalcone‐based compounds are of interest due to their diverse biological activities. This study aimed to synthesize and evaluate the proangiogenic potential of a series of novel chalcone derivatives and to elucidate their structure–activity relationship (SAR) using computational chemistry. We synthesized and tested six chalcone derivatives (1a–1f), with compound (*E*)‐1‐(3,4‐dimethoxyphenyl)‐3‐(3‐hydroxy‐4‐methoxyphenyl)prop‐2‐en‐1‐one (1c) exhibiting the highest activity. In the zebrafish model, treatment with 1c significantly increased the formation of branch points and vessel outgrowth in the subintestinal vein (SIV) (number of branch points: 1c group 2.28 ± 0.19, control group 0.79 ± 0.17; *p* < 0.001). Using Tg(*fli1:egfp*) transgenic zebrafish, we observed that 1c dramatically promoted the remodeling of the caudal vein plexus (CVP), leading to a significant increase in the number of intercapillary spaces (1c group 16.3 ± 1.9, mock group 11.6 ± 2). Mechanistically, real‐time polymerase chain reaction (RT‐PCR) results showed that 1c treatment upregulated the expression of key angiogenic genes cadherin 5 and neuropilin 1a, while downregulating the expression of fms‐related receptor tyrosine kinase 1. Furthermore, the chick embryo chorioallantoic membrane (CAM) assay confirmed that 1c effectively induced vascular network formation. Computational chemistry analyses (DFT, MESP, and FMO) were highly consistent with the biological activity. 1c possessed the highest electrophilicity index (*ω*), chemical potential (*μ*), and electron transfer capability (Δ*N*), as well as the strongest electrophilic site (*V*_*s*,max_), which explains its superior biological activity. This study confirms the potent proangiogenic activity of chalcone derivative 1c, providing a promising lead compound for the development of novel small‐molecule therapeutics targeting vascular dysfunction diseases.

## 1. Introduction

Angiogenesis, the process of new blood vessel growth from pre‐existing vasculature, is an indispensable process in physiological activities such as embryonic development, wound healing, and tissue repair. However, dysregulated angiogenesis is closely associated with the onset and progression of various diseases. For example, excessive angiogenesis is linked to cancer and diabetic retinopathy, whereas insufficient angiogenesis is implicated in ischemic diseases such as myocardial ischemia, stroke, and peripheral artery disease [1–6]. Therefore, the identification and development of chemical agents that can precisely modulate angiogenesis hold significant strategic importance for clinical therapy.

Several drugs that either promote or inhibit angiogenesis have been discovered. For example, Bevacizumab (Avastin), a monoclonal antibody that targets VEGF, along with small molecules such as sunitinib and sorafenib, which block angiogenesis‐related receptors, are FDA‐approved and used in the treatment of metastatic colorectal cancer, breast cancer, non–small cell lung cancer, and glioblastoma [7]. In contrast, proangiogenic agents are still relatively limited in number. To date, recombinant VEGF, angiopoietin‐1, the synthetic peptide vasculotide, and small molecules such as nicotine, salvianolic acid B, and compounds from traditional Chinese medicine are commonly used for treating ischemic diseases [8–11]. Despite these advancements, challenges such as drug resistance persist, and there is an ongoing need for novel, more effective proangiogenic agents, particularly small molecules.

Chalcone‐related compounds, which include both naturally occurring and synthetic molecules, are characterized by a backbone structure consisting of a benzene ring connected to an *α*,β‐unsaturated carbonyl group. These compounds have garnered significant attention due to their wide range of biological activities, including anticancer, anti‐inflammatory, antioxidant, and angiogenesis‐modulating effects [12–14]. For example, chalcone–thienopyrimidine and chalcone‐based phenothiazine derivatives have been shown to inhibit the growth of Hep G2 (hepatoma) and MCF‐7 (breast cancer) cells [15, 16]. Other chalcone derivatives containing a 2,4‐dichlorobenzenesulfonamide moiety have been reported to inhibit the proliferation of HeLa (cervical cancer) and HL60 (leukemia) cells [17]. These findings suggest that chalcone‐related compounds may be effective in inhibiting the growth of various cancer cell types.

In addition, several chalcone derivatives have demonstrated the ability to modulate angiogenesis. For instance, α‐fluorinated chalcones, butein (a plant‐based chalcone), and chalcone‐based 2,2‐dimethylbenzopyran have been reported to exhibit antiangiogenic effects [18–20], while others, including sulfonamide‐chalcone hybrids and dihydroxychalcones, have been shown to promote angiogenesis [21, 22]. These findings highlight the significant role chalcones play in regulating angiogenesis. Therefore, synthesizing and screening new chalcone‐based compounds could be a promising approach to discovering novel proangiogenic agents.

Several well‐established experimental methods are employed to study angiogenesis, such as the human umbilical vein endothelial cell (HUVEC) proliferation assay, corneal micropocket assay, zebrafish model, and chick chorioallantoic membrane (CAM) assay [23, 24]. In this study, we employed a systematic approach to design and synthesize six novel chalcone derivatives and evaluated and validated their activity using two complementary in vivo models: the zebrafish and the CAM assay. The zebrafish model is advantageous for its transparent embryos, external fertilization, and rapid development, making it particularly suitable for morphological analysis of vascular development (such as subintestinal vein (SIV) and caudal vein plexus (CVP)) [25]. The CAM assay provides validation of angiogenesis in a pseudomammalian extra‐embryonic environment. Furthermore, we utilized density functional theory (DFT) and molecular electrostatic potential (MESP) mapping to conduct an in‐depth computational chemical characterization of the active compound, aiming to establish the structure–activity relationship (SAR) and explain its superior biological activity at the molecular level. The ultimate goal of this study is to discover a novel small‐molecule proangiogenic agent with potential clinical application and to elucidate the underlying molecular and electronic structure basis.

## 2. Materials and Methods

### 2.1. Synthesis of New Chalcone Analogs

^1^H (300 MHz) and ^13^C (75 MHz) NMR spectra were recorded on a Varian Mercury‐300 NMR spectrometer (Agilent, Santa Clara, CA, USA). Chemical shifts downfield from tetramethylsilane (TMS), which was used as an internal reference, were reported on the *δ* scale as parts per million (ppm.) and were attributed to variations in the electronic environment surrounding the nuclei. Mass spectra were measured using a VG Analytical Model 70–250 s mass spectrometer (Varian, Palo Alto, CA, USA). All reagents were used as obtained commercially.

(*E*)‐1‐(3,4‐dimethoxyphenyl)‐3‐(3‐isopropoxy‐4‐methoxyphenyl)prop‐2‐en‐1‐one (1a) ^1^H‐NMR (CDCl_3_): *δ* 7.77 (1H, d, *J* = 15.6 Hz), 7.65 (1H, dd, *J* = 8.4, 2.1 Hz), 7.62 (1H, dd, *J* = 8.1, 1.8 Hz), 7.43 (1H, d, *J* = 15.6 Hz), 7.17 (1H, d, *J* = 1.8 Hz), 6.93 (1H, d, *J* = 8.4 Hz), 6.91 (1H, d, *J* = 8.4 Hz), 4.68 (1H, septa, *J* = 6.0 Hz), 3.96 (6H, s), 3.95 (3H, s), 3.94 (3H, s), 1.43 (6H, d, *J* = 6.0 Hz); ^13^C‐NMR (CDCl_3_): *δ* 188.6, 151.6, 151.2, 150.0, 149.2, 143.9, 131.2, 128.0, 122.8, 122.7, 119.7, 112.8, 111.4, 111.1, 110.1, 71.2, 56.1, 55.9 (2C), 21.9; HRMS Calcd for C_21_H_24_O_5_: 356.1624. Found: 356.1628 [26].

(*E*)‐1‐(3,4‐dimethoxyphenyl)‐3‐(3‐methoxy‐4‐hydroxyphenyl)prop‐2‐en‐1‐one (1b) ^1^H‐NMR (CDCl_3_): *δ* 7.75 (1H, d, *J* = 15.6 Hz), 7.68 (1H, d, *J* = 7.8 Hz), 7.44 (1H, d, *J* = 15.6 Hz), 7.26 (1H, s), 7.24 (1H, d, *J* = 8.4 Hz), 7.13 (1H, s), 6.97–6.93 (2H, m), 5.71 (1H, s), 3.99 (3H, s), 3.98 (3H, s), 3.96 (3H, s); ^13^C‐NMR (CDCl_3_): *δ* 188.6, 153.1, 149.2, 148.7, 145.9, 143.9, 131.5, 128.7, 122.9, 122.7, 119.8, 112.9, 110.7, 110.6, 109.9, 76.7, 56.1, 56.0; HRMS Calcd for C_18_H_18_O_5_: 314.1154. Found: 314.1160 [26].

(*E*)‐1‐(3,4‐dimethoxyphenyl)‐3‐(3‐hydroxy‐4‐methoxyphenyl)prop‐2‐en‐1‐one (1c) ^1^H‐NMR (CDCl_3_): *δ* 7.75 (1H, d, *J* = 15.5 Hz), 7.69 (1H, dd, *J* = 8.4, 2.0 Hz), 7.64 (1H, d, *J* = 1.96 Hz), 7.44 (1H, d, *J* = 15.5 Hz), 7.32 (1H, d, *J* = 2.1 Hz), 7.15 (1H, dd, *J* = 8.4, 2.0 Hz), 6.95 (1H, d, *J* = 8.4 Hz), 6.89 (1H, d, *J* = 8.3 Hz), 5.71 (1H, s), 3.99 (3H, s), 3.98 (3H, s), 3.96 (3H, s); ^13^C‐NMR (CDCl_3_): *δ* 188.6, 153.1, 149.2, 148.7, 145.9, 143.9, 131.5, 128.7, 122.9, 122.7, 119.8, 112.9, 110.7, 110.6, 109.9, 76.7, 56.1, 56.0; HRMS Calcd for C_18_H_18_O_5_: 314.1154. Found: 314.1158 [27].

(*E*)‐1‐(3,4‐diisopropoxyphenyl)‐3‐(4‐isopropoxy‐3‐methoxyphenyl)prop‐2‐en‐1‐one (1d) ^1^H‐NMR (CDCl_3_): *δ* 7.74 (1H, d, *J* = 15.6 Hz), 7.65 (1H, d, *J* = 8.4 Hz), 7.64 (1H, s), 7.38 (1H, d, *J* = 15.6 Hz), 7.20 (1H, d, *J* = 8.4 Hz), 7.16 (1H, s), 6.95 (1H, d, *J* = 8.4 Hz), 6.90 (1H, d, *J* = 8.4 Hz), 4.62 (2H, septa, *J* = 6.0 Hz), 4.45 (1H, septa, *J* = 6.0 Hz), 3.92 (3H, s), 1.40 (3H, d, *J* = 6.0 Hz), 1.39 (3H, d, *J* = 6.0 Hz), 1.36 (3H, d, *J* = 6.0 Hz); ^13^C‐NMR (CDCl_3_): *δ* 188.9, 153.6, 150.2, 149.7, 148.5, 144.1, 131.6, 128.0, 123.4, 122.7, 119.7, 118.1, 115.0, 114.5, 110.9, 72.6, 71.7, 71.3, 56.1, 22.2, 22.1, 22.0; HRMS Calcd for C_25_H_32_O_5_: 412.2250. Found: 412.2252 [27].

(*E*)‐1‐(3,4‐dimethoxyphenyl)‐3‐(4‐isopropoxy‐3‐methoxyphenyl)prop‐2‐en‐1‐one (1e) ^1^H‐NMR (CDCl_3_): *δ* 7.76 (1H, d, *J* = 15.6 Hz), 7.68 (1H, dd, *J* = 8.7, 1.2 Hz), 7.62 (1H, d, *J* = 1.8 Hz), 7.41 (1H, d, *J* = 15.6 Hz), 7.22 (1H, d, *J* = 8.7 Hz), 6.94 (1H, d, *J* = 8.4 Hz), 6.91 (1H, d, *J* = 8.4 Hz), 4.62 (1H, septa, *J* = 6.0 Hz), 3.97 (6H, s), 3.93 (3H, s), 1.41 (6H, d, *J* = 6.0 Hz); ^13^C‐NMR (CDCl_3_): *δ* 188.7, 150.3, 149.8, 149.2, 144.2, 131.6, 128.0, 123.5, 122.8, 122.7, 119.6, 114.6, 111.1, 110.9, 109.9, 71.3, 56.1, 22.0; HRMS Calcd for C_21_H_24_O_5_: 356.1624. Found: 356.1630 [28].

(*E*)‐1,3‐bis(4‐isopropoxy‐3‐methoxyphenyl)prop‐2‐en‐1‐one (1f) ^1^H‐NMR (CDCl_3_): *δ* 7.76 (1H, d, *J* = 15.6 Hz), 7.67–7.62 (2H, m), 7.41 (1H, d, *J* = 15.6 Hz), 7.22 (1H, dd, *J* = 2.1, 8.1 Hz), 7.17 (1H, d, *J* = 2.1 Hz), 6.94 (1H, d, *J* = 8.1 Hz), 6.91 (1H, d, *J* = 8.1 Hz), 4.64 (1H, septa, *J* = 6.0 Hz), 4.62 (1H, septa, *J* = 6.3 Hz), 3.95 (3H, s), 6.93 (3H, s), 1.43 (6H, d, *J* = 6.0 Hz), 1.41 (6H, d, *J* = 6.3 Hz); ^13^C‐NMR (CDCl_3_): *δ* 188.7, 150.3, 149.8, 149.2, 144.2, 131.6, 128.0, 123.5, 122.8, 122.7, 119.6, 114.6, 111.1, 110.9, 109.9, 71.7, 71.3, 56.1 (2C), 22.1, 22.0; HRMS Calcd for C_23_H_28_O_5_: 384.1937. Found: 384.1941 [28]. All the NMR information is listed in the supporting data (please see Figures S1–S6).

### 2.2. Computational Details

Chalcone and chalcone‐functionalized compounds 1a–f are optimized using DFT calculations at the M06‐2X/6–311 + G(d, p) level of theory [29]. The subsequent harmonic vibrational analysis of the optimized structure ensures the existence of real frequency in the structure belongs to the minima on the potential energy surface (PES). Grimme’s dispersion correction D3 coefficients are used to incorporate the long‐range intramolecular interactions [30]. The choice of Minnesota M06‐2X functional is a global hybrid with 54% Hartree–Fock exchange well documented for main group thermochemistry, kinetics, and noncovalent interactions. All DFT calculations are performed using the Gaussian G16 Rev. C.01 program package [31].

When a chemical reaction takes place, the highest occupied molecular orbital (HOMO) and the lowest unoccupied molecular orbital (LUMO) are the major contributing electronic orbitals. The HOMO embodies electron donation, while the LUMO is involved in the acceptance of electrons. Thus, probing the frontier molecular orbital (FMO) analysis, i.e., the energy gap between the LUMO–HOMO, is an important indicator for understanding intramolecular charge transfer owing to its tendency to measure electrical conductivity. Molecules with a lower energy gap are treated as polarizable molecules with high chemical reactivity and low kinetic stability, and such molecules are often pronounced as soft molecules [32]. A molecule with a large energy gap is considered a hard molecule. According to Koopmann’s theorem, HOMO energy (*E*_HOMO_) is related to ionization potential (*I*), while LUMO energy (*E*_LUMO_) is the electron affinity (*A*) [33]. The energy gap (Δ*E*_gap_) between the HOMO and LUMO implies the resistance to both the redox reaction and low chemical reactivity of the molecular systems. Besides the Δ*E*_gap_, the quantum chemical descriptors that include chemical potential (*μ*), chemical hardness (*η*), electrophilicity index (*ω*), and softness (*σ*), and the number of transferred electrons (Δ*N*) are evaluated using the following equations [34]:

(1) I=−EHOMO,

(2) A=−ELUMO,

(3) μ=−χ=I+A2≈EHOMO+ELUMO2,

(4) η=I−A≈EHOMO−ELUMO,

(5) ω=μ22η≈EHOMO+ELUMO24ELUMO−EHOMO=I+A24A−I,

(6) σ=1I−A=1η≈1ELUMO−EHOMO,

(7) ΔN=−μη.

The real space visualization of the three‐dimensional (3D) molecular electrostatic surface potential (MESP) *V*(*r*) data derived from the optimized structure using wave function analysis‐surface analysis suite (WFA‐SAS) was used for the interpretation and prediction of chemical reactivity of the molecular systems [35]. The quantitative potential was achieved by the rigorous mapping of the MESP topology achieved by computing the gradients of electrostatic potential (∇*V*(*r*)) and the Hessian matrix elements at the critical points (CPs), where ∇*V*(*r*) = 0. In the MESP topology, the bonded regions, i.e., the inter‐/intramolecular, show a characteristic of (3, −1) bond critical points (BCPs), while the high electron density regions, such as lone pair and *π*‐delocalized regions, show (3, +3) CPs with negative potential, *V*_min_. The analysis of *V*_min_ provides a powerful and worthwhile descriptor to characterize the high electron density regions in a molecular system, as it corresponds to the localized information of the wave function at this point, influenced by the nuclei and electronic distribution according to Coulomb’s law. The analysis of *V*_min_ successfully explains the chemical reactivity and measures the substituent effects. *V*_min_ at the lone pair region of the small molecule ligand has been used as a reliable electronic parameter to assess the *σ*‐donating ability to the complementary receptor environment.

### 2.3. Experimental Animals

Wild‐type AB strain zebrafish and green fluorescent blood vessel transgenic fish Tg(*fli1:egfp*) were maintained as described previously [21, 25]. Chick embryos were purchased from a local farm (Chu‐Lin Company, Chiao Hsi, I‐Lan, Taiwan). All animal studies and procedures were approved by Tamkang University’s Biosafety Committee (Approval No: AZ‐BS‐111001).

### 2.4. Drug Exposure and Survival Rate Analysis

Wild‐type zebrafish embryos were collected and divided into groups of 100 embryos each. Each group was treated with one of six different compounds (1a–1f) at concentrations of 1, 3, 5, 10, or 15 mg/L. The “no treatment” group was incubated in water, and the “mock” group was incubated in 0.1% DMSO. The exposure period was from 48 h postfertilization (hpf) to 72 hpf. Survival rates were assessed at 72 hpf by counting live embryos, and the data were analyzed using Excel software. All experiments were performed in triplicate, and results are presented as average ± standard deviation (SD).

### 2.5. Alkaline Phosphatase Staining

Alkaline phosphatase staining was performed to visualize endothelial cells in blood vessels, following previously established protocols [21]. Briefly, embryos were fixed at 72 hpf in 4% paraformaldehyde and washed with PBS (containing 10% Tween 20). Alkaline phosphatase activity was detected using an NBT/BCIP substrate (Merck), and vascular growth patterns were recorded. Data were analyzed using Excel and presented as average ± SD, with experiments conducted in triplicate.

### 2.6. Fluorescent Blood Vessel Growth Pattern Recording

The transgenic zebrafish line Tg(*fli1:egfp*), which expresses GFP in blood vessels, was used to study vascular growth. Embryos were divided into groups (25 embryos/group), treated with either 0.1% DMSO (Mock) or 5 mg/L of 1c, from 12 to 36 hpf. At 36 hpf, the number of intercapillary spaces in the CVP was counted. Data were analyzed using Excel and presented as average ± SD, with all experiments conducted in triplicate.

### 2.7. Real‐Time Polymerase Chain Reaction (RT‐PCR)

RNA extraction, cDNA synthesis, and RT‐PCR were carried out as previously described [11]. Primer sequences used to detect the expression of *β-actin* (internal control), *flt1*, *cdh5*, and *nrp1a* were synthesized as follows:

 
*β-actin*: forward: 5′‐CGAGCAGGAGATGGGAACC‐3′,
 reverse: 5′CAACGGAAACGCTCATTGC‐3′;
 
*flt1*: forward: 5′‐AACTCACAGACCAGTGAACAAGA‐3′,
 reverse: 5′‐TTAGCCTTCTGTGGGTATGTCCA‐3′;
 
*cdh5*: forward: 5′‐GGTGCCTCCGACAAGGATGA‐3′,
 reverse: 5′‐AACACTCTTTTGCTCTGGCGT‐3′;
 
*nrp1a*: forward: 5′‐CTCCAACAAACCCTACCAGGT‐3′,
 reverse: 5′‐TCGGTGATGTCCACCATGATTTC‐3′.

### 2.8. CAM Assay

Chick embryos at 9 days postfertilization (35 HH) were used for the CAM assay. The eggshells were carefully removed to expose the CAM. Circular filter papers (2.55 mm in radius) were soaked with either DMSO (mock) or 100 mg/L 1c for 10 min and then placed on the membrane. The embryos were sealed with 3M tape and incubated at 37°C. By 12 days postfertilization (38 HH), the photos were taken using an iPhone 7. The area inside a big circle from each embryo was observed for vascular growth patterns, and the number of branch points was counted by AngioTool.

### 2.9. Statistical Analysis

**FIGURE 1:**
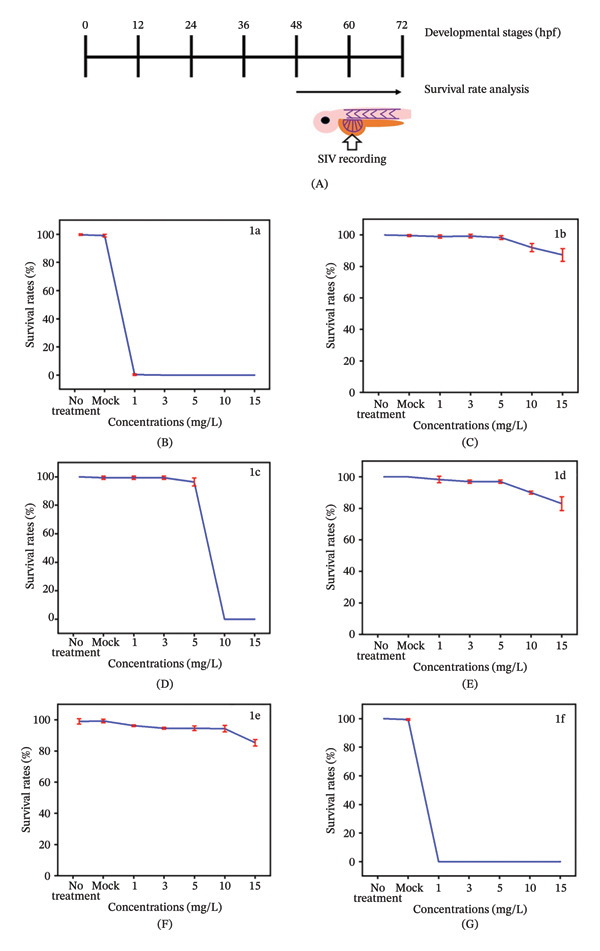
Exposure method and survival rate analysis of chalcone‐based chemicals. Zebrafish embryos developed at 48 h postfertilization (hpf) were collected, randomly divided into 100 embryos per experimental group, and soaked in water (no treatment), 0.1% DMSO (mock), or 1, 3, 5, 10, and 15 mg/L of test chemicals (compounds 1a–1f) for 24 h. By 72 hpf, survival embryos were counted, and their survival rates were calculated. (A) Exposure is from 48 to 72 hpf. (B–G) Survival rate analysis for each chalcone‐based chemical.

**FIGURE 2:**
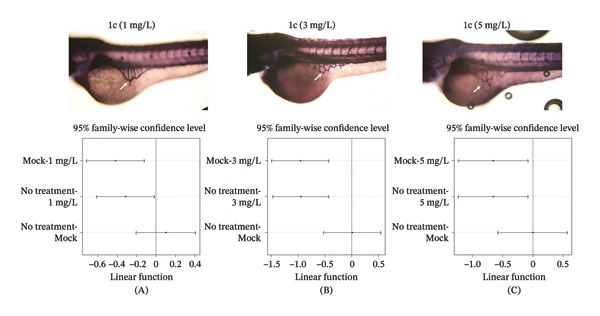
Recording of SIV outgrowth in compound 1c‐exposed embryos. (A–C) Representative images of AP‐stained embryos (AB strain) treated with compound 1c (1, 3, and 5 mg/L) from 48 to 72 hpf. Embryos at 72 hpf were subjected to alkaline phosphatase staining. White arrows marked the positions of SIV outgrowth. Data were subjected to statistical analysis using one‐way ANOVA and the Tukey–Kramer honestly significant difference (HSD) test.

**FIGURE 3:**
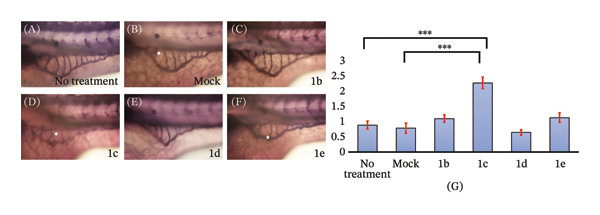
The SIV branch point recording of chalcone‐based compounds on the zebrafish. (A–F) Representative images of AP‐stained embryos (AB strain) treated with water only (no treatment), 0.1% DMSO (mock), or water containing chalcone‐based chemicals (compounds 1b, 1c, 1d and 1e) from 48 to 72 hpf. Embryos at 72 hpf were subjected to alkaline phosphatase staining. Stars marked the positions of SIV branch points. (G) Data were subjected to statistical analysis using Excel T.TEST (^∗∗∗^ *p* < 0.001).

**FIGURE 4:**
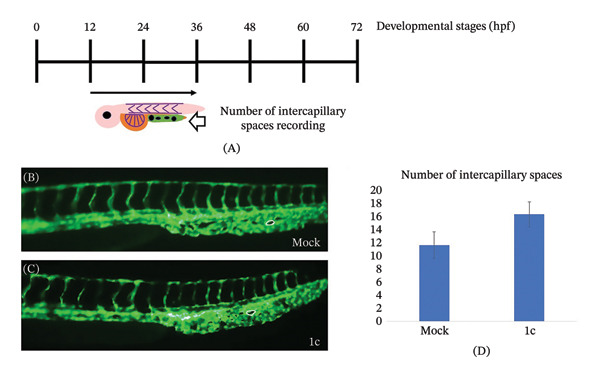
Compound 1c exposure increased the number of intercapillary spaces in the CVPs. (A–C) Exposure is from 12 to 36 hpf. Representative pictures showing the CVPs of Tg(*fli1:egfp*) mock control and compound 1c‐treated embryos at 36 hpf. (D) Statistical figures of the number of intercapillary spaces in each group. The *X* and *Y* axes represent the average number of intercapillary spaces and each experimental group, respectively. (^∗∗^ *p* < 0.01).

**FIGURE 5:**
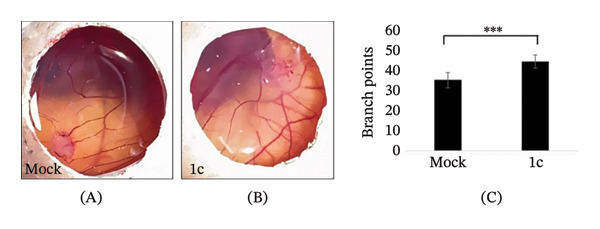
CAM assay analysis of compound 1c‐exposed chick embryos. The 12‐day postfertilization (38 HH) chick embryos were placed with (A) a DMSO‐soaked filter or (B) a 100 mg/L compound 1c‐treated filter. The area inside each embryo was observed for vascular growth pattern recording. (C) Statistical figures of the number of branch points in each group. The number of branch points was counted by AngioTool [36] and was analyzed using Excel. The *X* and *Y* axes represent the average number of branch points and each experimental group, respectively (^∗∗∗^ *p* < 0.001).

**FIGURE 6:**
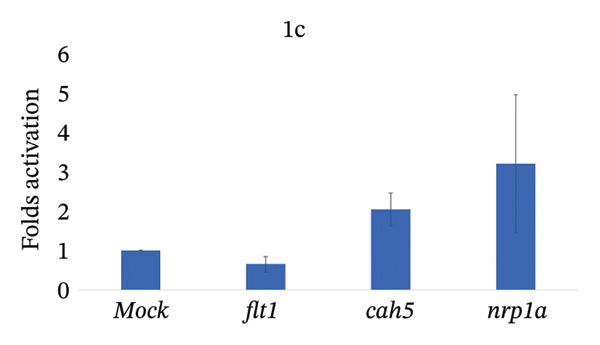
Compound 1c exposure affects the expressions of blood vessel growth‐related genes, *cdh5*, *nrp1a*, and *flt1*. Relative quantification of gene expressions using the comparative CT method (CT: cycles of real‐time PCR; relative folds to control group = 2^−ΔΔCT^; the GMD‐treated group is significantly different from the corresponding mock group).

All statistical analyses were performed using the R package Rcmdr (R Version 4.3.2) and Microsoft Excel. One‐way analysis of variance (ANOVA) was used to assess differences among treatment groups. When the overall ANOVA was significant, pairwise comparisons were made using the Tukey–Kramer honestly significant difference (HSD) test. Survival rate analyses, ANOVA, and the Tukey–Kramer HSD test for the results presented in Figures 1 and 2 were implemented using the Rcmdr package. For the branch points and CVP analyses shown in Figures 3, 4, and 5, results are presented as mean ± SD, and two‐sample *t*‐tests were performed using Microsoft Excel. A *p* value of < 0.05 was considered statistically significant. For multiple comparisons, the family‐wise error rate was controlled at 0.05 using the Tukey–Kramer adjustment. In addition, the sample sizes (*n*) for the analyses in Figures 1, 2, 3, 4, and 5 refer to the number of biological replicates (*n*), and those for the analysis in Figure 6 refer to technical replicates (*N*).

## 3. Results

### 3.1. Chemistry

**FIGURE 7:**
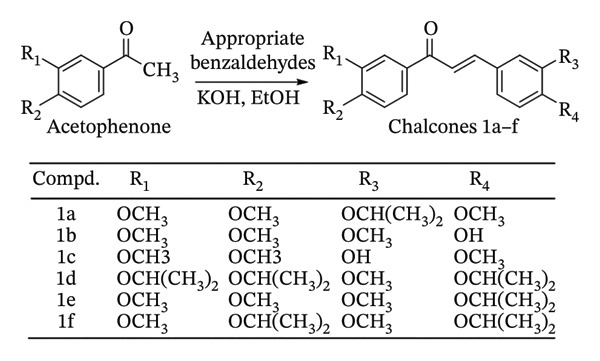
Structures of chalcone‐based compounds used in this study. Six compounds were synthesized (compounds 1a–1f). Their chemical formula, exact mass, and molecular weight are listed below each compound.

In this study, we synthesized six chalcone‐based compounds (1a–1f) using a procedure similar to that described previously [37]. The synthesis of O‐isopropyl‐acetophenone or its aldehyde derivative was straightforward. Acetophenone or the aldehyde was initially reacted with isopropyl bromide and potassium carbonate in DMF, yielding excellent results. The resulting acetophenone was then reacted with the corresponding benzaldehydes in the presence of 5N KOH, leading to the formation of products 1a‐1f with isolated yields ranging from 76% to 90% (Figure 7). Analysis of the ^1^H‐NMR spectra confirmed that the chalcone‐based compounds were in the trans configuration, with *J*_*H* *α*,*H* *β*_ values ranging from 15 to 16 Hz. The NMR spectra for compounds 1a–1f are provided in the supporting data (Figures S1–S6).

### 3.2. Survival Rate Analysis of Chalcone‐Based Compounds in Zebrafish Embryos

In this study, zebrafish embryos were exposed to varying concentrations (0–15 mg/L) of chalcone‐based compounds from 48 to 72 hpf (Figure 1A), and their survival rates were recorded. The results showed very low survival rates (0%–0.33% ± 0.58, *n* = 100, triplicate repeats) in embryos exposed to 1–15 mg/L of 1a and 1f (Figure 1B,E). In contrast, survival rates in the 1–15 mg/L exposure groups for 1b, 1d, and 1e were significantly higher, ranging from 83% ± 4.36 to 99.33% ± 1.15 (*n* = 100) (Figure 1C,E,F). Similarly, the “no treatment” and “mock” control groups showed near‐complete survival rates (99% ± 1–100%, *n* = 100) (Figure 1B–G). Particularly, embryos exposed to lower concentrations (1, 3, and 5 mg/L) of 1c exhibited high survival rates (96.33% ± 2.89–99.33% ± 1.15, *n* = 100). However, no surviving embryos were observed at higher concentrations (10 and 15 mg/L) of 1c (Figure 1D). These findings suggest that 5 mg/L of chalcone‐based compounds (except for 1a and 1f) is the optimal concentration for subsequent experiments in the zebrafish model.

### 3.3. Effects of Chalcone‐Based Compounds on SIV Growth

In zebrafish embryos, the SIV forms as a simple vascular network early in development, undergoing significant branching and outgrowth between 48 and 72 hpf. To study the effects of chalcone‐based compounds on vascular development, we examined SIV branch point formation after exposure to 1b–1e (5 mg/L) using AP‐staining (Figure 3A–F). Exposure to 1c led to a significant increase in SIV branch point formation, with an average of 2.28 ± 0.19 branch points (*n* = 68), compared to 0.89 ± 0.13 in the “no treatment” group (*n* = 58) and 0.79 ± 0.17 in the “mock” group (*n* = 35) (Figure 3G). Statistical analysis using a *t*‐test confirmed significant differences between the “1c” and “no treatment” (*p* < 0.001) or “mock” groups (*p* < 0.001).

We further investigated the effect of different concentrations of 1c (1, 3, and 5 mg/L) on SIV outgrowth (Figure 2A), white arrows indicate outgrowth). The mean counts of SIV outgrowth in the “no treatment,” “mock,” and treated groups were as follows: 0.22 ± 0.08 (*n* = 58), 0.12 ± 0.04 (*n* = 58), 0.54 ± 0.12 (*n* = 65), 1.59 ± 0.20 (*n* = 68), and 1.03 ± 0.24 (*n* = 29), respectively. One‐way ANOVA showed significant differences (*p* values: 0.0028, < 0.0001, and 0.0094 for 1, 3, and 5 mg/L vs. controls). Post hoc Tukey–Kramer HSD testing revealed that all treated groups (1, 3, and 5 mg/L) had significantly higher SIV outgrowth counts compared to the control groups (“no treatment” and “mock”) (Figure 2).

### 3.4. Effects of 1c on CVP Remodeling

The zebrafish CVP consists of a network of capillaries, and intercapillary spaces are commonly used as indicators of proper vascular remodeling during angiogenesis [38]. We assessed the effect of 1c on CVP remodeling by counting these intercapillary spaces. The average number of intercapillary spaces in the “mock” and 1c‐treated groups was 11.6 ± 2 (*n* = 25) and 16.3 ± 1.9 (*n* = 25), respectively (Figure 4). These findings suggest that 1c treatment promoted remodeling of the CVP by increasing the number of intercapillary spaces.

### 3.5. 1c Alters Expression of Angiogenesis‐Related Genes

To investigate the molecular mechanisms underlying the angiogenic effects of 1c, we performed RT‐PCR to measure the expression of three key blood vessel growth‐related genes: cadherin 5 (*cdh5*), neuropilin 1a (*nrp1a*), and fms‐related receptor tyrosine kinase 1 (*flt1*) [24, 38, 39]. Figure 6 shows that the expression levels of *cdh*5 and *nrp*1a were significantly elevated in the 1c‐treated group (2.04 ± 0.42 and 3.20 ± 1.76, respectively, *N* = 3) compared to the mock control. In contrast, the expression of *flt1* was significantly reduced (0.65 ± 0.19, *N* = 3) in the 1c‐treated group (Figure 6). These results suggest that 1c modulates angiogenesis by influencing key factors involved in cell–cell adhesion (*cdh5*), coreceptor signaling (*nrp1a*), and VEGF receptor signaling (*flt1*), supporting its proangiogenic activity.

### 3.6. 1c Induces Blood Vessel Growth in Chick Embryos

To further confirm the proangiogenic effects of 1c, we utilized a chick embryo model with the CAM assay, a well‐established method for assessing vascular growth. The results demonstrated that, compared to mock control embryos, regions treated with 1c displayed a denser and more extensive vascular network within the circled area. Notably, additional vascular branches extended from the main vessels (Figure 5C, 35.25 ± 3.72 vs. 44.58 ± 3.32, *n* = 12; Figure mock vs. 1c‐treated). These findings validate that 1c enhances blood vessel formation in a chick embryo model, reinforcing its angiogenic potential.

### 3.7. Chemical Reactivity of the Compounds

**FIGURE 8:**
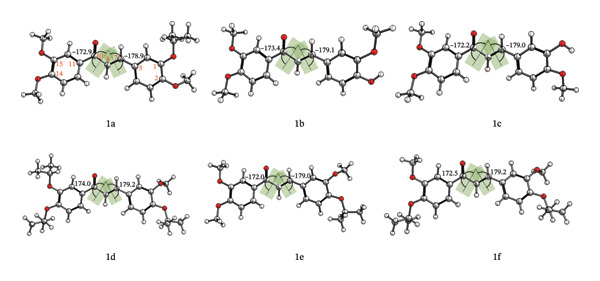
Optimized molecular geometries and dihedral angles of chalcone analogs (compounds 1a–1f). The figure shows the energy‐minimized structures of six chalcone analogs (1a–1f), highlighting their near‐planar configurations and trans orientation across the conjugated double bond. Dihedral angles along C11–C10–C8–C7 and C5–C7–C8–C10 are annotated to evaluate structural planarity. Among them, compound 1a shows the highest deviation (ca. 7°) from planarity, whereas the rest adopt nearly planar geometries with deviations less than 2°. These conformational features suggest differential reactivity profiles influenced by substituent positions and electronic effects on the aromatic rings.

From the geometry optimization, it is found that the molecular structures are near‐planar and adopt a *trans* configuration with respect to the conjugated C=C double bond. The measured dihedral plane along C11–C10–C8–C7 and C5–C7–C8–C10 reveals that the former deviates by ca. 7° from planarity, attributed to the maximum nonplanarity, while the latter deviates by only ca. 2° to adopt a near‐planar configuration (Figure 8).

**FIGURE 9:**
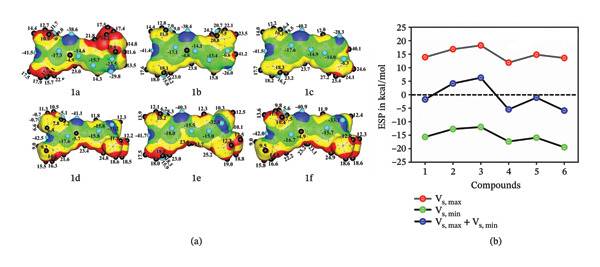
Electrostatic surface potential (ESP) analysis of chalcone analogs (compounds 1a–1f). (a) Mapped ESP surfaces of each compound are shown on an isodensity surface of 0.001 electrons/Bohr^3^. Red and blue regions represent areas of highest positive (*V*_*s*,max_) and negative (*V*_*s*,min_) electrostatic potential, indicating potential electrophilic and nucleophilic sites, respectively. *V*_*s*,max_ is mainly located on the electron‐donating substituents (‐OCH_3_, ‐O(CH_3_)_2_), while *V*_*s*,min_ is concentrated on carbonyl oxygens and ‐OH groups. (b) Quantitative plot of *V*_*s*,max_, *V*_*s*,min_, and their sum [∑(*V*_*s*,max_ + *V*_*s*,min_)] for all six compounds. Compound 1c exhibits the strongest electrophilic character, while compound 1f shows the strongest nucleophilic nature. These ESP descriptors highlight 1c and 1f as the most chemically reactive among the series.

The electron‐donating substituent groups ‐OH, ‐OCH_3_, and ‐O(CH_3_)_2_ at the terminal phenyl ring of positions C1, C2, C14, and C15 influence imposed reactivity. The substituents exhibit a negative inductive (−*I*) effect in the order of ‐O(CH_3_)_2_ > ‐OH > ‐OCH_3_, which play a pivotal role in the structural reactivity of the chalcone‐functionalized compounds. To discern the reactivity, we mapped MESPs on the molecular structure (Figure 9a). The positive potential (*V*_*s*,max_) and the negative potential (*V*_*s*,min_), represented in red and blue, respectively, highlighting the electrophilic and nucleophilic regions in space, are important descriptors in determining the reactivity. The *V*_*s*,max_ regions are present predominantly on the electron‐donating substituents ‐OCH_3_ and ‐O(CH_3_)_2_ at the meta and para positions of the phenyl ring. The *V*_*s*,min_ regions are located on the carbonyl oxygen and the ‐OH substituted compounds 1b and 1c. While the highest *V*_*s*,min_ with values of ca. −41.4–−42.5 kcal/mol are seen at the center of the two oxygen atoms of the substituents ‐OCH_3_ and ‐O(CH_3_)_2_, the carbonyl oxygen atoms have *V*_*s*,min_ of ca. −38.4–−41.1 kcal/mol. In contrast, the *V*_*s*,max_ values are well distributed on the molecular surfaces. The highest average *V*_*s*,max_ (ca. 18.18 kcal/mol) observed for 1c portrays its stronger tendency to attract nucleophiles, while 1d has least *V*_*s*,max_ (ca. 11.89 kcal/mol). Furthermore, while 1c has the weakest nucleophilic site (*V*_*s*,min_ = ca. −11.96 kcal/mol), 1f has the strongest nucleophilic site (*V*_*s*,min_ = −19.48 kcal/mol). Furthermore, the net average potential, *V*_avg_ = ∑(*V*_*s*,max_ + *V*_*s*,min_), depicts that 1b (4.17 kcal/mol) and 1c (6.31 kcal/mol) have strong electrophilic sites. While 1a (−1.73 kcal/mol), 1d (−5.44 kcal/mol), 1e (−1.08 kcal/mol), and 1f (−5.88 kcal/mol) show a nucleophilic character, 1f shows the strongest nucleophilic character (Figure 9). Thus, MESP suggests that 1c and 1f are prone to maximum reactivity, as their *V*_*s*,max_ and *V*_*s*,min_ values, respectively, are strongest in nature compared to their respective congeners.

### 3.8. FMO Analysis

**FIGURE 10:**
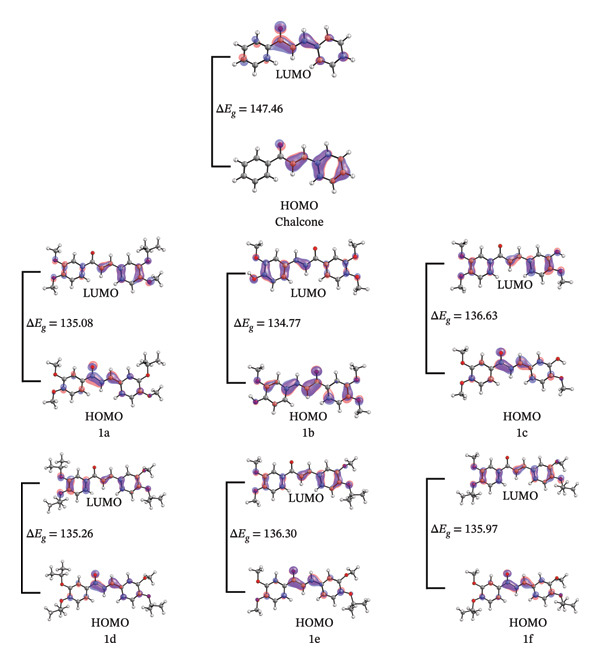
Frontier molecular orbital analysis of chalcone analogs (compounds 1a–1f). The highest occupied molecular orbital (HOMO) and lowest unoccupied molecular orbital (LUMO) are mapped on the electron density isosurface of 0.05 a.u., and the energy differences (Δ*E*_*g*_) between the LUMO and HOMO are given in kcal/mol.

FMO analysis provides crucial insights into the electronic structure and chemical reactivity of the chalcone derivatives studied. The HOMO in these molecules is delocalized over the conjugated *π* system encompassing the aromatic rings and *α*,β‐unsaturated carbonyl moiety, indicating a strong *π* character and suggesting potential for electron donation. In contrast, the LUMO is primarily localized on the carbonyl oxygen, highlighting this region as the principal electrophilic site susceptible to nucleophilic attack (Figure 10). The computed Δ*E*_*g*_ values, ranging from 134.77 kcal/mol (1b, lowest) to 136.63 kcal/mol (1c, highest), are all significantly lower than that of pristine chalcone (147.46 kcal/mol), suggesting increased reactivity with moderate to high molecular stability. Electron‐donating substituents such as ‐OH, ‐OCH_3_, and ‐O(CH_3_)_2_, when positioned at the meta and para locations, exert a +M (resonance donating) effect but also an inductive electron‐withdrawing (−*I*) effect due to the electronegativity of oxygen. The resonance effect is diminished at the meta position, allowing the −*I* effect to dominate, making these groups weakly deactivating overall. Consequently, the order of reactivity based on electronic effects follows −OH > −OCH_3_ > −O(CH_3_)_2_. This trend explains why compounds 1d and 1f, which contain more ‐O(CH_3_)_2_ groups, exhibit higher Δ*E*_*g*_ values and reduced reactivity. Despite 1c showing the highest Δ*E*_*g*_, its electronic chemical potential (*μ* = 97.31 kcal/mol), the highest among the series, indicates a greater tendency to donate electrons, enhancing its nucleophilic character. Moreover, the electrophilicity index (*ω* ≈ 34.59 kcal/mol for 1a–1c) is higher than that of pristine chalcone, implying a greater ability to accept electrons, thus acting as better electrophiles. Compound 1c also exhibits the highest electron transfer parameter (Δ*N*), reinforcing its dual reactivity profile (Table 1). Taken together, these findings suggest that 1c, despite its marginally higher energy gap, possesses the most favorable balance of nucleophilicity and electrophilicity, making it a promising candidate for complexation with electrophilic biomolecular targets such as proteins.

**TABLE 1:** Highest occupied molecular orbital energy (*E*_HOMO_), lowest unoccupied molecular orbital energy (*E*_LUMO_), ionization energy (*I*), electron affinity (*A*), energy gap (Δ*E*_gap_), chemical potential (*μ*), hardness (*η*), electrophilicity index (*ω*), softness (*σ*), and maximum number of electrons (Δ*N*) that an electrophile can acquire.

Comp.	*E*_HOMO_	*E*_LUMO_	*I*	*A*	*μ*	*η*	*ω*	*σ*	Δ*N*
1a	−164.59	−29.52	164.59	29.52	97.05	135.08	34.87	3.94	−0.72
1b	−164.20	−29.42	164.20	29.42	96.81	134.77	34.77	3.95	−0.72
1c	−165.54	−28.90	165.54	28.90	97.22	136.63	34.59	3.89	−0.71
1d	−162.82	−27.56	162.82	27.56	95.19	135.26	33.50	3.93	−0.70
1e	−164.79	−28.49	164.79	28.49	96.64	136.30	34.26	3.90	−0.71
1e	−163.83	−27.86	163.83	27.86	95.85	135.97	33.78	3.91	−0.7
Chalcone	−183.42	−35.95	183.42	35.95	109.68	147.46	40.79	3.61	−0.74

*Note:* All the energy values are reported in units of kcal/mol.

## 4. Discussion

In this study, we identified a synthetic chalcone derivative, (*E*)‐1‐(3,4‐dimethoxyphenyl)‐3‐(3‐hydroxy‐4‐methoxyphenyl)prop‐2‐en‐1‐one (compound 1c), as a novel proangiogenic agent with validated activity in both zebrafish and chick embryo models. Chalcones have been widely investigated for their anti‐inflammatory, antioxidant, and anticancer properties [40–42], yet their potential in promoting angiogenesis has been underexplored. Our findings expand the therapeutic landscape of this chemical class by demonstrating that 1c effectively induces blood vessel outgrowth during early developmental stages.

Angiogenesis plays a central role in both physiological processes, such as tissue regeneration, and pathological conditions, including cancer and chronic inflammation. As previously noted [6], excessive angiogenesis is implicated in tumor growth and inflammatory diseases, whereas insufficient angiogenesis contributes to ischemic disorders. The modulation of angiogenesis has therefore become a key therapeutic strategy, as emphasized previously [7], underscoring the value of developing small‐molecule agents such as 1c.

In zebrafish embryos, treatment with 1c enhanced intersegmental vessel formation, while the CAM assay showed increased vascular branching. These phenotypic changes were accompanied by the upregulation of angiogenesis‐related genes, including *cdh5* and *nrp1a*, alongside downregulation of *flt1* [25, 43]. Since *flt1* encodes a decoy VEGF receptor, its suppression may increase VEGF‐A availability for Kdrl (VEGFR2), thereby amplifying proangiogenic signaling [25].

A key structural insight from our analysis lies in the substitution pattern of the hydroxyl and methoxy groups on the B ring of the chalcone scaffold. Compound 1c differs from its positional isomer 1b only in the orientation of these two groups, yet displayed markedly higher angiogenic efficacy. Both functional groups are chemically stable under physiological pH (6.8–7.4), which is consistent across zebrafish embryos and chick CAM. Therefore, the divergent biological activity is unlikely due to stability, but rather to spatial configuration. This observation highlights a precise SAR, where even subtle changes in substituent positioning significantly affect biological function [36, 44]. Such knowledge provides a valuable starting point for rational design and optimization of chalcone‐based angiogenic agents.

While these findings are promising, certain limitations remain. The current study did not include protein‐level validation (e.g., VEGFR2 phosphorylation or downstream signaling), and mammalian models were not employed to assess systemic efficacy or safety. Furthermore, the pharmacokinetic and toxicological profiles of 1c have yet to be elucidated. Addressing these issues will be crucial for the translation of 1c into potential therapeutic applications.

In conclusion, this work introduces a novel chalcone derivative with robust proangiogenic activity, validated across two complementary in vivo models. The computational analyses (MESP and FMO) show trends that are consistent with the experimentally observed in vivo activity, where compound 1c exhibits superior antiangiogenic effects. Specifically, the MESP surface of compound 1c displays the most pronounced positive electrostatic potential regions (*V*_*s*,max_), suggesting an enhanced propensity to interact with nucleophilic residues at the binding interface. This observation is further supported by FMO‐derived descriptors, including its relatively higher chemical potential (*μ*) and electron transfer capacity (Δ*N*), indicating increased electronic reactivity. Importantly, the in vivo results establish the biological efficacy of compound 1c, while the computed electronic descriptors provide a mechanistic rationale for its enhanced activity. We therefore interpret electrophilicity and related parameters as supporting contributors within a broader SAR, rather than as sole determinants of biological function. In contrast, 1f exhibited stronger nucleophilicity but lower overall reactivity, consistent with its moderate in vivo response. Thus, 1c emerges as a promising candidate for further biological evaluation. By integrating morphological assays, gene expression profiling, and structural analysis, our study contributes to the expanding field of small‐molecule modulators of angiogenesis and supports future development of 1c analogs as candidate therapeutics for vascular insufficiency.

## Funding

This work was supported by the National Science and Technology Council, Grant No. 114‐2113‐M‐032‐003.

## Conflicts of Interest

The authors declare no conflicts of interest.

## Supporting Information

Additional supporting information can be found online in the Supporting Information section.

## Supporting information


**Supporting Information** The ^1^H NMR spectra of compounds 1a–1f (Figures S1–S6) were consistent with their proposed structures. Diagnostic signals corresponding to aromatic protons (*δ* 6.8–7.8 ppm), *α*,β‐unsaturated carbonyl moieties (*δ* 7.2–7.7 ppm), methoxy groups (*δ* 3.9–4.0 ppm), and isopropoxy substituents (*δ* 4.6–4.7 and 1.3–1.6 ppm) were clearly observed. These spectral data confirmed the successful synthesis of the target chalcone derivatives.

## Data Availability

The data that support the findings of this study are available from the corresponding author upon reasonable request.
