# Supplementary figures and images for: A Phenotype‐Driven Discovery of Pro‐Revascularization Chalcone Derivatives Using Zebrafish and CAM Models
Source: J Toxicol. 2026 Jul 12;2026:6939974. doi: 10.1155/jt/6939974 (PMC13358358; doi:10.1155/jt/6939974)

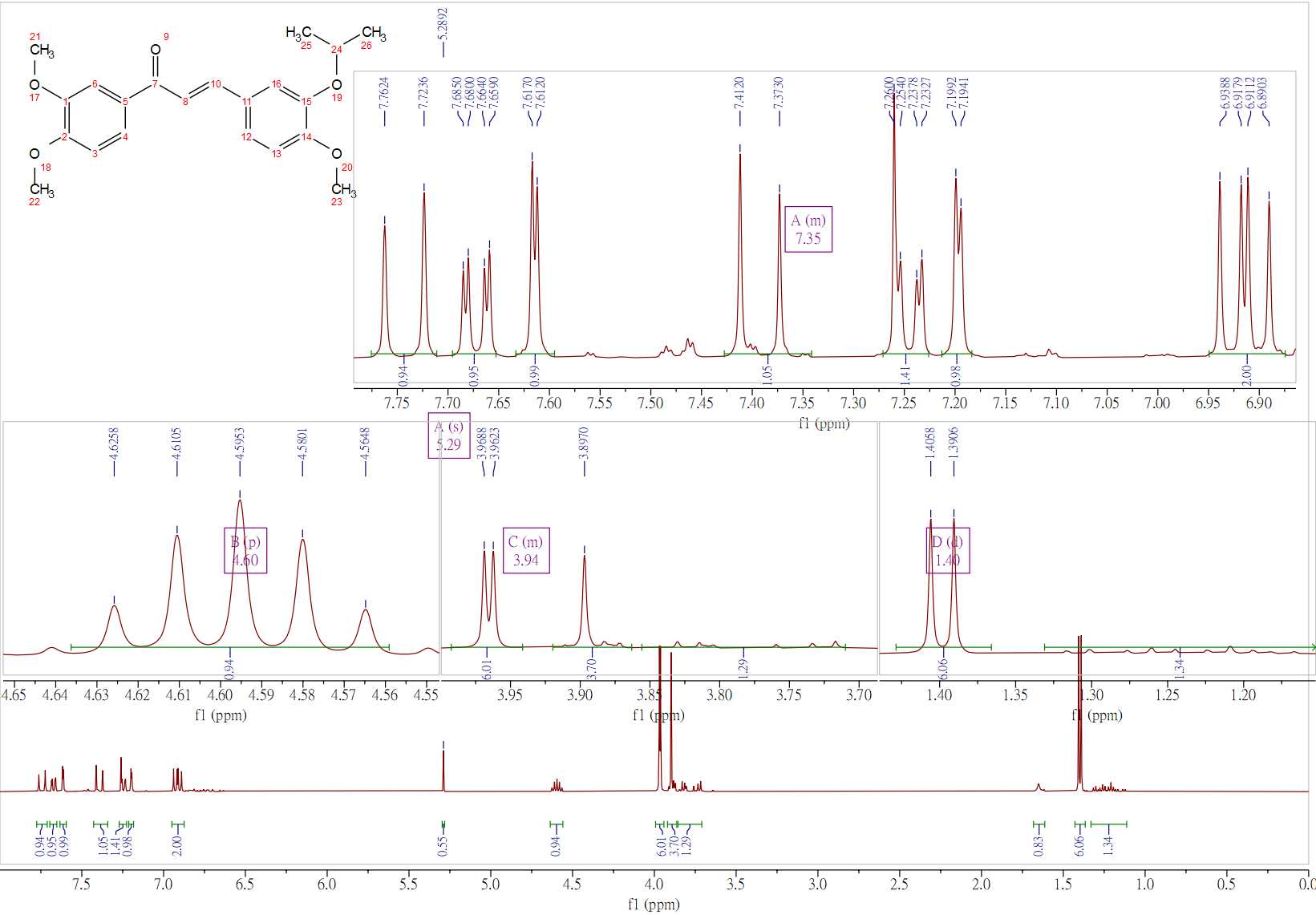

Supplement: Supplementary file 1 — Supporting Information The 1H NMR spectra of compounds 1a–1f (Figures S1–S6) were consistent with their proposed structures. Diagnostic signals corresponding to aromatic protons (δ 6.8–7.8 ppm), α,β‐unsaturated carbonyl moieties (δ 7.2–7.7 ppm), methoxy groups (δ 3.9–4.0 ppm), and isopropoxy substituents (δ 4.6–4.7 and 1.3–1.6 ppm) were clearly observed. These spectral data confirmed the successful synthesis of the target chalcone derivatives. [file JT-2026-6939974-s001.zip › Fig S1 1a.jpg]

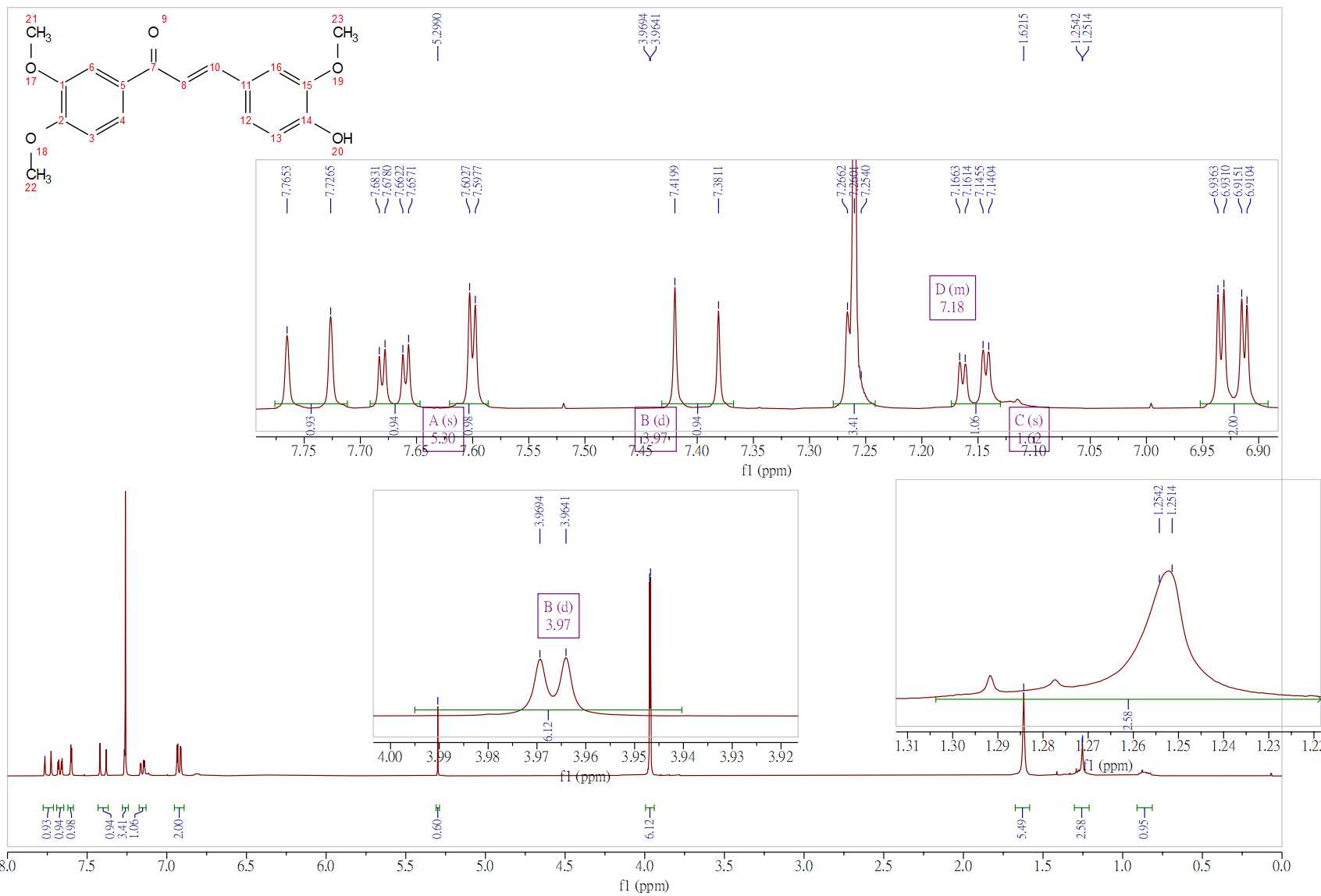

Supplement: Supplementary file 1 — Supporting Information The 1H NMR spectra of compounds 1a–1f (Figures S1–S6) were consistent with their proposed structures. Diagnostic signals corresponding to aromatic protons (δ 6.8–7.8 ppm), α,β‐unsaturated carbonyl moieties (δ 7.2–7.7 ppm), methoxy groups (δ 3.9–4.0 ppm), and isopropoxy substituents (δ 4.6–4.7 and 1.3–1.6 ppm) were clearly observed. These spectral data confirmed the successful synthesis of the target chalcone derivatives. [file JT-2026-6939974-s001.zip › Fig S2 1b.jpg]

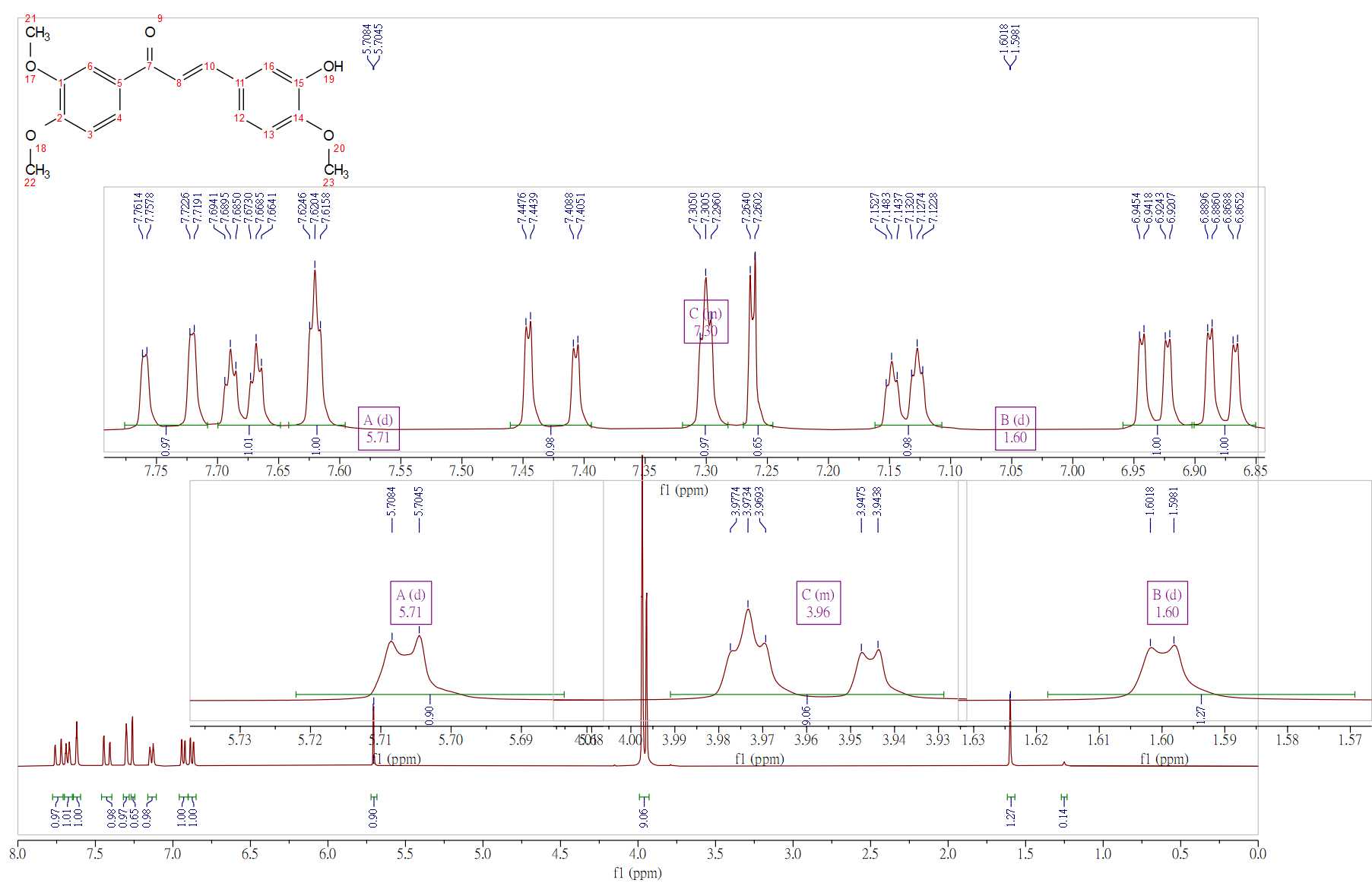

Supplement: Supplementary file 1 — Supporting Information The 1H NMR spectra of compounds 1a–1f (Figures S1–S6) were consistent with their proposed structures. Diagnostic signals corresponding to aromatic protons (δ 6.8–7.8 ppm), α,β‐unsaturated carbonyl moieties (δ 7.2–7.7 ppm), methoxy groups (δ 3.9–4.0 ppm), and isopropoxy substituents (δ 4.6–4.7 and 1.3–1.6 ppm) were clearly observed. These spectral data confirmed the successful synthesis of the target chalcone derivatives. [file JT-2026-6939974-s001.zip › Fig S3 1c.jpg]

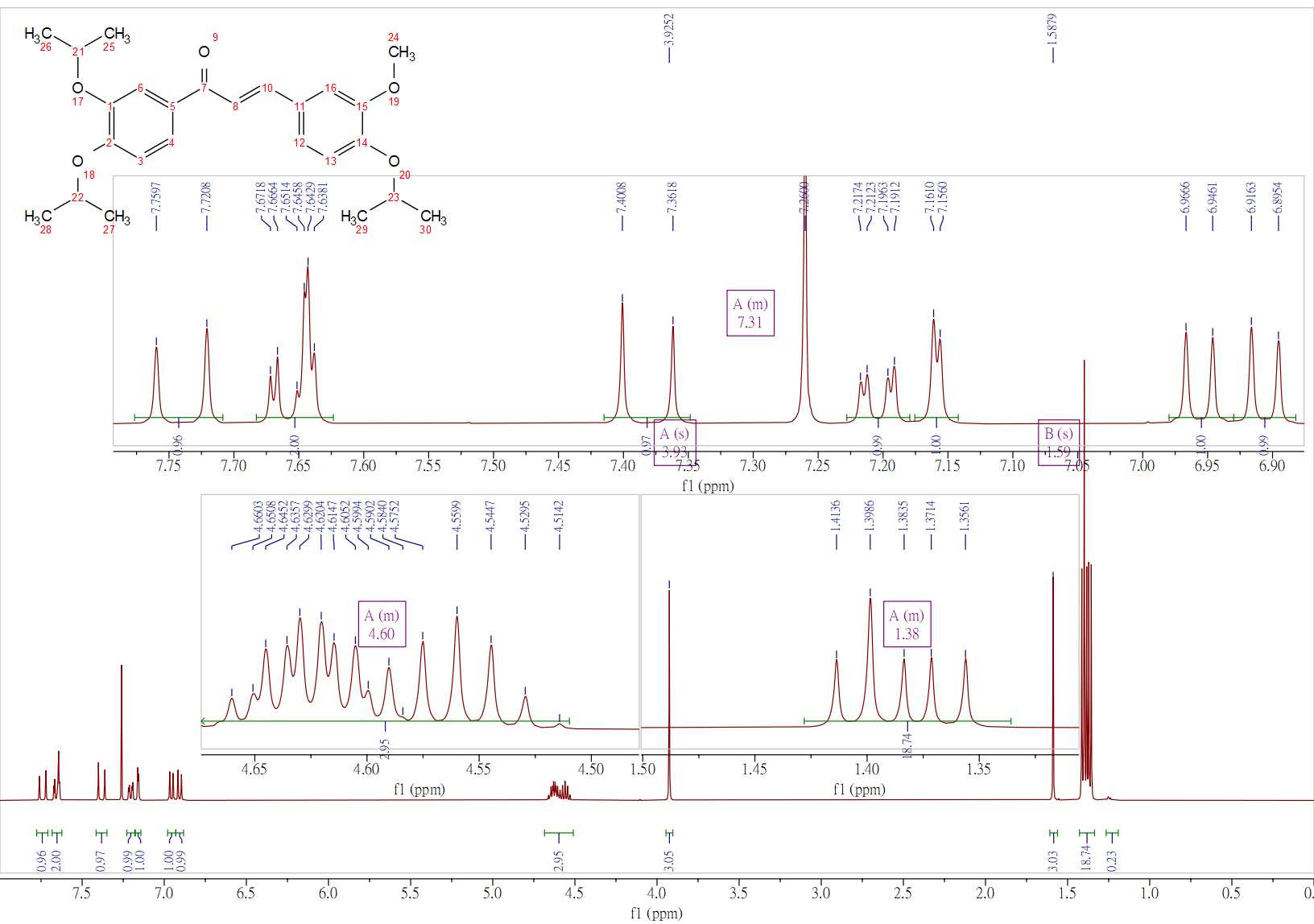

Supplement: Supplementary file 1 — Supporting Information The 1H NMR spectra of compounds 1a–1f (Figures S1–S6) were consistent with their proposed structures. Diagnostic signals corresponding to aromatic protons (δ 6.8–7.8 ppm), α,β‐unsaturated carbonyl moieties (δ 7.2–7.7 ppm), methoxy groups (δ 3.9–4.0 ppm), and isopropoxy substituents (δ 4.6–4.7 and 1.3–1.6 ppm) were clearly observed. These spectral data confirmed the successful synthesis of the target chalcone derivatives. [file JT-2026-6939974-s001.zip › Fig S4 1d.jpg]

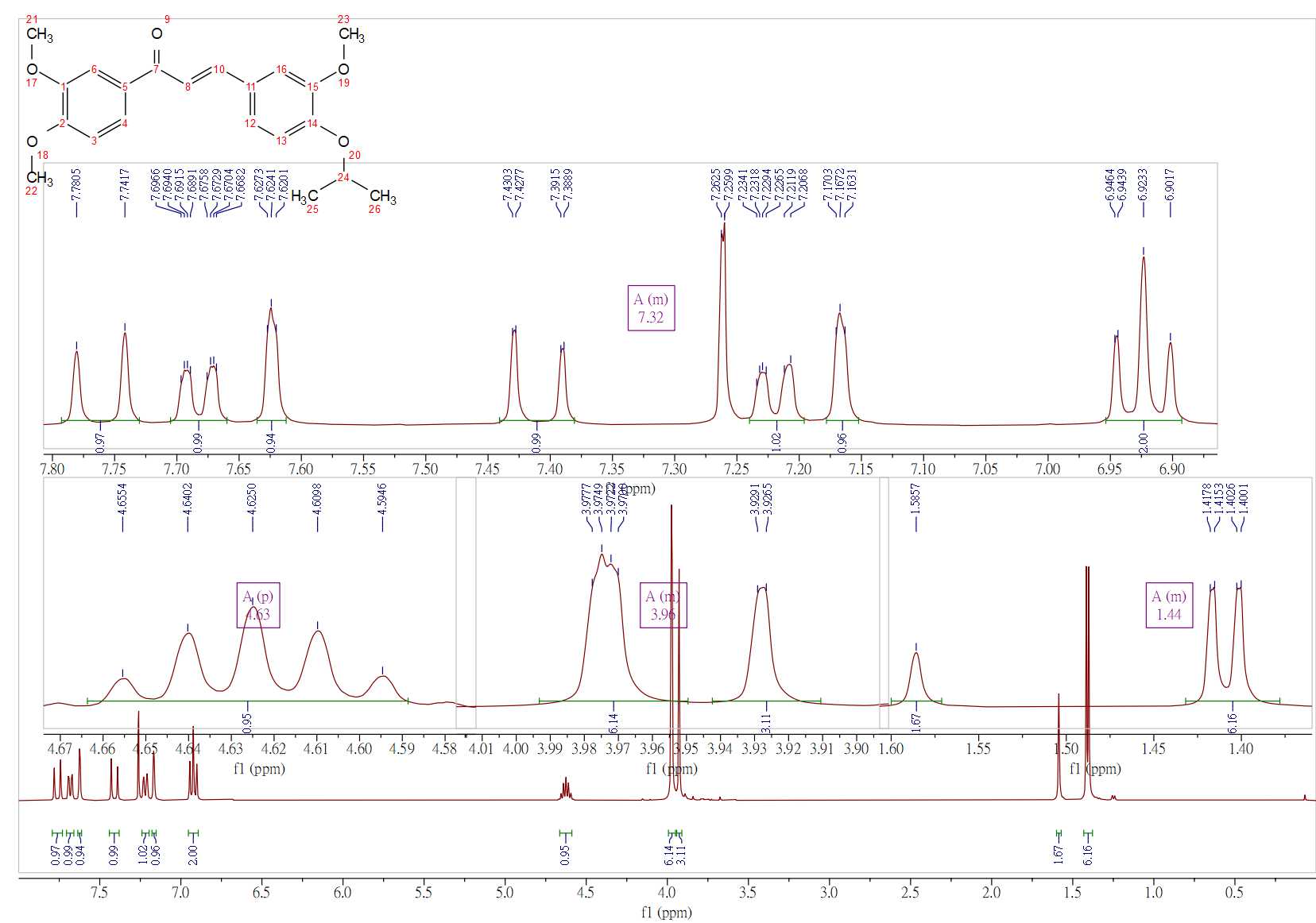

Supplement: Supplementary file 1 — Supporting Information The 1H NMR spectra of compounds 1a–1f (Figures S1–S6) were consistent with their proposed structures. Diagnostic signals corresponding to aromatic protons (δ 6.8–7.8 ppm), α,β‐unsaturated carbonyl moieties (δ 7.2–7.7 ppm), methoxy groups (δ 3.9–4.0 ppm), and isopropoxy substituents (δ 4.6–4.7 and 1.3–1.6 ppm) were clearly observed. These spectral data confirmed the successful synthesis of the target chalcone derivatives. [file JT-2026-6939974-s001.zip › Fig S5 1e.jpg]

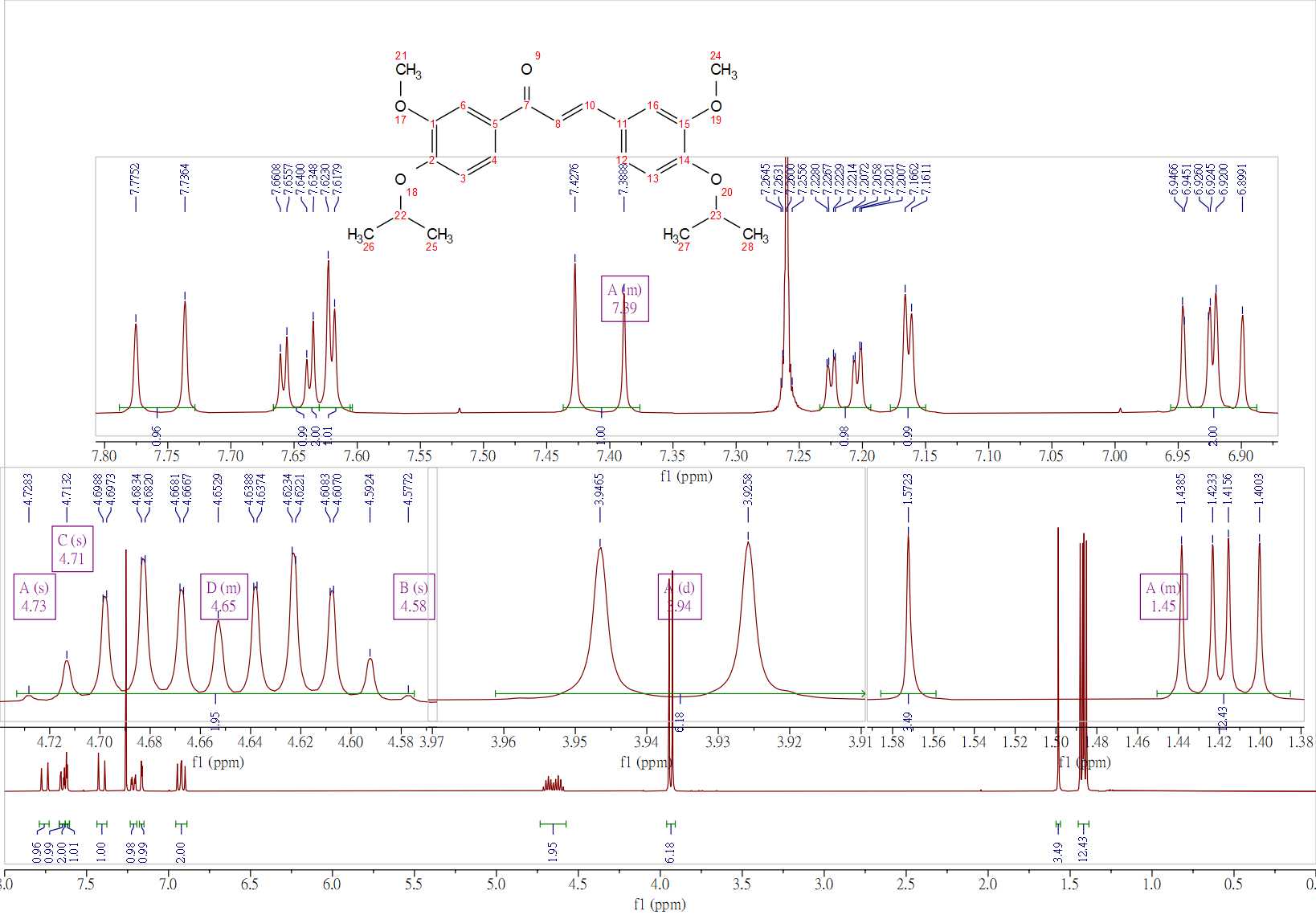

Supplement: Supplementary file 1 — Supporting Information The 1H NMR spectra of compounds 1a–1f (Figures S1–S6) were consistent with their proposed structures. Diagnostic signals corresponding to aromatic protons (δ 6.8–7.8 ppm), α,β‐unsaturated carbonyl moieties (δ 7.2–7.7 ppm), methoxy groups (δ 3.9–4.0 ppm), and isopropoxy substituents (δ 4.6–4.7 and 1.3–1.6 ppm) were clearly observed. These spectral data confirmed the successful synthesis of the target chalcone derivatives. [file JT-2026-6939974-s001.zip › Fig S6 1f.jpg]
